# ProCKSI: a decision support system for Protein (Structure) Comparison, Knowledge, Similarity and Information

**DOI:** 10.1186/1471-2105-8-416

**Published:** 2007-10-26

**Authors:** Daniel Barthel, Jonathan D Hirst, Jacek Błażewicz, Edmund K Burke, Natalio Krasnogor

**Affiliations:** 1 ASAP, School of Computer Science and IT, University of Nottingham, Nottingham, NG8 1BB, UK; 2 School of Chemistry, University of Nottingham, Nottingham, NG7 2RD, UK; 3 Institute of Bioorganic Chemistry, Polish Academy of Sciences, 61-704 Poznan, Poland; 4 The Institute of Computing Science, 60-965 Poznan, Poland

## Abstract

**Background:**

We introduce the decision support system for *Protein (Structure) Comparison, Knowledge, Similarity and Information *(*ProCKSI*). ProCKSI integrates various protein similarity measures through an easy to use interface that allows the comparison of multiple proteins simultaneously. It employs the *Universal Similarity Metric *(USM), the *Maximum Contact Map Overlap *(MaxCMO) of protein structures and other external methods such as the *DaliLite *and the *TM-align *methods, the *Combinatorial Extension *(CE) of the optimal path, and the *FAST Align and Search Tool *(FAST). Additionally, ProCKSI allows the user to upload a user-defined similarity matrix supplementing the methods mentioned, and computes a similarity consensus in order to provide a rich, integrated, multicriteria view of large datasets of protein structures.

**Results:**

We present ProCKSI's architecture and workflow describing its intuitive user interface, and show its potential on three distinct test-cases. In the first case, ProCKSI is used to evaluate the results of a previous CASP competition, assessing the similarity of proposed models for given targets where the structures could have a large deviation from one another. To perform this type of comparison reliably, we introduce a new consensus method. The second study deals with the verification of a classification scheme for protein kinases, originally derived by *sequence *comparison by Hanks and Hunter, but here we use a consensus similarity measure based on *structures*. In the third experiment using the Rost and Sander dataset (RS126), we investigate how a combination of different sets of similarity measures influences the quality and performance of ProCKSI's new consensus measure. ProCKSI performs well with all three datasets, showing its potential for complex, simultaneous multi-method assessment of structural similarity in large protein datasets. Furthermore, combining different similarity measures is usually more robust than relying on one single, unique measure.

**Conclusion:**

Based on a diverse set of similarity measures, ProCKSI computes a consensus similarity profile for the entire protein set. All results can be clustered, visualised, analysed and easily compared with each other through a simple and intuitive interface.

ProCKSI is publicly available at  for academic and non-commercial use.

## Background

An important theme within structural bioinformatics is the analysis of protein sequences, the assessment of protein structural similarities and the inference of their biological functions. All of these play crucial roles in drug design and other structural inference activities [1] such as homology modeling and protein structure prediction. There, it is important to evaluate similarity among a large number of structures and to identify similar predictions or find the closest prediction to a given target [2].

Structural comparison and clustering is challenging, and effective algorithms continue to be introduced. The simplest global measure for protein structure comparison is the root mean square deviation (RMSD) [3,4]. More sophisticated methods are fragment matching [5,6], geometric hashing [7], comparison of distance matrices [8], Monte Carlo (MC) algorithms or simulated annealing [8], maximum sub-graph detection [9], local geometry matching [10], incremental combinatorial extension (CE) of the optimal path [11], local global alignment (LGA) [12], dynamic programming [13-15], genetic algorithms (GA) [16], consensus shapes [17] or consensus structures [18], contact map overlaps (CMO) [19-25]), secondary structure matching (SSM) [26], memetic algorithms [27], or maximum clique detection [28,29]. In addition to these algorithms comparing two rigid protein structures, methods for flexible structure alignment have also been developed [30-32].

Many databases and web servers have been introduced implementing different concepts and aspects of the methodologies described above. An overview of recommended, well-tested resources, tools and databases for protein 3D structure and sequence comparison is given in the *Bioinformatics Links Directory *[33]. For more detailed information, the reader is referred to the overview articles of Galparin [34-36], and the webserver issues [37,38] and database issues [39-42] in Nucleic Acids Research.

### ProCKSI's Philosophy

As it is evident from the list above, there are many biologically meaningful definitions of protein similarity. Several methods have been proposed and there is a variety of structure classification servers and databases available, each of them with its own interface, philosophy and, most importantly, biological conception of what "similarity" means. Paradoxically, the availability of all these methods with their unique interfaces and underlying biological hypotheses makes it *more*, and not *less *difficult for a structural biologist to decide which method to apply in which cases. Moreover, it is common to find papers related to protein structure comparison where the authors claim that their new method is better than another on a small set of test cases. These types of comparison can be misleading in at least two ways. First, changing the algorithm used to compare structures often inadvertently introduces a different comparison *criterion*, hence changing the problem itself. Secondly, the comparisons are usually done on a reduced number of data sets with characteristics that make them suitable to the new (implicit) criterion. In this paper we take the view that there is not one problem of protein structural comparison but rather many different, yet related, structural similarity problems where each of them might be best tackled with a different method. Hence, attempting to find *the best method *for protein structure comparison is a chimera. Instead, in line with other recent suggestions [43,44] that the integration of a variety of feature detection techniques could enhance protein comparison, we advocate here an integrative approach that harnesses the best in each available method. This change in philosophy allows us to treat the assessment of protein structure comparisons as a *decision support *problem in which the task of the bioinformatician is to build up computer facilities that empower the user to make an informed decision with the minimum possible overhead. The advantage of this viewpoint is that it does not call for the abolition of one method in favour of another one but rather for the intelligent integration of every possible protein structure comparison method into one unified tool.

### ProCKSI's Core Protocol

In this paper, we take the first steps towards the creation of an intelligent decision support system for protein structure comparison. We introduce a new meta-server for ***Pro****tein (Structure) ****C****omparison*, ***K****nowledge*, ***S****imilarity, and ****I****nformation *(ProCKSI) implementing the protocol, published by Krasnogor and Pelta [24], and substantially extending it. This server facilitates protein structural comparison by allowing the user to compare multiple protein structures seamlessly using multiple similarity methods through a unique and integrated interface. As ProCKSI adheres to the philosophy mentioned above where different conceptions of similarities can be used under different circumstances, it deals well with comparisons of both very divergent structures and quite similar ones. Until now, methods were proposed that work well in either of these cases but not in both simultaneously. The first case is dealt with using the top level of the protocol, namely, the *Universal Similarity Metric *(USM, [45]) and the latter case by means of the *Maximum Contact Map Overlap *(MaxCMO) method [20,21,23]. As it has been shown in other contexts (e.g. protein structure prediction) that meta-servers sometimes outperform human experts [46,47], the similarity results returned by these two methods can also be complemented with other comparison methods, and even integrated into a *consensus *similarity assessment. Hence, motivated by the observations above and in recognition that a) in many situations a very detailed comparison is needed and b) biologists may also want to compare a protein set from several viewpoints or conceptions of similarity simultaneously, ProCKSI harvests results from well-established external protein comparison servers and methods. It makes them available and readily comparable with one another, and combines the various similarity measures to give a consensus similarity profile for a given dataset.

The first level of similarity assessment utilises the USM as a similarity measure between two protein structures *s*_1 _and *s*_2_. Their contact map representations are then used to approximate heuristically the Kolmogorov complexity of the proteins, comparing their information content. The approximation of the Kolmogorov complexity is done using a compression algorithm (e.g. compress, gzip, bzip2, ppmz2). The pairwise similarities are then expressed as the *Normalised Compression Distance *(see Eq. 1), NCD, where *K*(*s*_*i*_) represents the Kolmogorov complexity of object *s*_*i *_and where *K*(*s*_*i*_|*s*_*j*_) is the conditional complexity. NCD is a very effective universal, i.e. problem-domain independent, similarity metric particularly with distantly related structures [24] and sequences [48].

NCD(s1,s2)=max{K(s1|s2),K(s2|s1)}max{K(s1),K(s2)}
 MathType@MTEF@5@5@+=feaafiart1ev1aaatCvAUfKttLearuWrP9MDH5MBPbIqV92AaeXatLxBI9gBaebbnrfifHhDYfgasaacH8akY=wiFfYdH8Gipec8Eeeu0xXdbba9frFj0=OqFfea0dXdd9vqai=hGuQ8kuc9pgc9s8qqaq=dirpe0xb9q8qiLsFr0=vr0=vr0dc8meaabaqaciaacaGaaeqabaqabeGadaaakeaacqWGobGtcqWGdbWqcqWGebarcqGGOaakcqWGZbWCdaWgaaWcbaGaeGymaedabeaakiabcYcaSiabdohaZnaaBaaaleaacqaIYaGmaeqaaOGaeiykaKIaeyypa0ZaaSaaaeaacqWGTbqBcqWGHbqycqWG4baEcqGG7bWEcqWGlbWscqGGOaakcqWGZbWCdaWgaaWcbaGaeGymaedabeaakiabcYha8jabdohaZnaaBaaaleaacqaIYaGmaeqaaOGaeiykaKIaeiilaWIaem4saSKaeiikaGIaem4Cam3aaSbaaSqaaiabikdaYaqabaGccqGG8baFcqWGZbWCdaWgaaWcbaGaeGymaedabeaakiabcMcaPiabc2ha9bqaaiabd2gaTjabdggaHjabdIha4jabcUha7jabdUealjabcIcaOiabdohaZnaaBaaaleaacqaIXaqmaeqaaOGaeiykaKIaeiilaWIaem4saSKaeiikaGIaem4Cam3aaSbaaSqaaiabikdaYaqabaGccqGGPaqkcqGG9bqFaaaaaa@669B@

As the USM is a very general metric, for more fine grained comparisons ProCKSI implements a metaheuristic to compute the *Maximum Contact Map Overlap *(MaxCMO) of pairs of proteins counting the number of equivalent residues (alignments) and additionally the number of equivalent contacts (overlaps). Under the MaxCMO model, an amino acid residue *a*_1 _from one protein is aligned to an amino acid residue *a*_2 _from a second protein if a contact of *a*_1 _in the first protein (*C*(*a*_1_)) can also be aligned to a contact of *a*_2 _in the second protein (*C*(*a*_2_)) closing a cycle of size 4 in the graph representation of the contact map. A further restriction for the overlaps is that they should not produce crossing edges. That is, if *a*_1 _is aligned to *a*_2_, *C*(*a*_1_) is aligned to *C*(*a*_2_) and, without loss of generality, *a*_1 _<*C*(*a*_1_) (i.e. the atom or residue *a*_1 _appears before than *C*(*a*_1_) in the sequence) then *a*_2 _<*C*(*a*_2_). Thus, an overlap in this model is a strong indication of topological similarity between the pair of proteins as it takes into consideration the local environment of each of the aligned residues. In addition to the two methods previously described, ProCKSI utilises the DaliLite workbench [8,49] and the Combinatorial Extension (CE) method [11], both providing the statistical significance of an alignment (Z-score), the TM-align method [50] using TM-scores, and the FAST method [28] providing SN-scores as their key similarity measure.

The results are analysed with standard clustering methods. The clusters thus obtained can be visualised using either a linear, a circular or a hyperbolic representation of the hierarchical similarity tree [51] that captures the dataset's structural organisation. Additional analysis tools permit the comparison and integration of multiple similarity measures, so as to give a *consensus *similarity cluster. The analysis tools cannot only be used with ProCKSI's results but also in combination with additional similarity matrices that the user provides. Through this mechanism, the set of similarity matrices can be extended by *any *similarity measure that the user deems to be important allowing one to refine the similarity consensus for a given dataset. It allows the user to add different information which is not produced by the methods currently integrated with ProCKSI.

In addition to its core protocol for protein comparison as described above, ProCKSI aims to give an overall picture of the protein universe by providing, for each protein in the dataset, as much information and knowledge as possible. To this end, ProCKSI directly links to the PDB repository [52], the *Structural Classification of Proteins *(SCOP) database [53,54], and the *Protein Structure Classification *(CATH) database [55,56]. Sometimes, it might be useful not only to know more about the structure itself but also to get information about the literature where a certain protein occurs. ProCKSI therefore links to the *information hyperlinked over proteins *(iHOP) service [57,58] providing an interactive network of proteins within the related literature.

In the next section we show the general architecture and workflow of ProCKSI and explain in detail the features introduced above.

## Implementation

**Figure 1:**
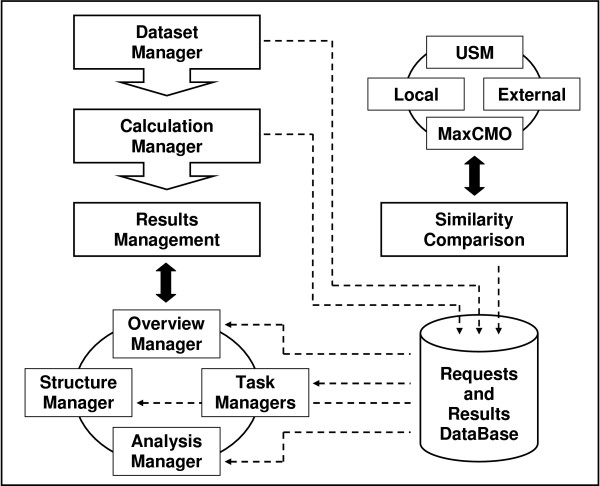
**ProCKSI's Architecture and Workflow**. Overview over the workflow and architecture of ProCKSI with its multiple similarity comparison methods: Universal Similarity Metric (USM), Maximum Contact Map Overlap (MaxCMO), and other local and external methods. Currently, these are the DaliLite and TM-align methods, the Combinatorial Extension (CE) of the optimal path, and the FAST Align and Search Tool (FAST).

ProCKSI's workflow (Figure 1) consists of three main stages: *Dataset Management*, *Calculation Management *and *Results Management*. The latter includes the following parts: *Overview Management*, *Structure Management*, *Analysis Management *and the special *Task Management *that is associated with each of the different similarity comparison methods. In what follows we describe the functionality of each of these components and how they inter-operate.

### Dataset Management

The server can handle protein structure files in the *Protein Data Bank *(PDB) format, which can be downloaded directly from the PDB repository [52] by entering the PDB codes of the proteins. Alternatively, they can be uploaded from the user's local hard disc sequentially, i.e. one protein file at a time, or as archives (TAR, ZIP) containing multiple protein structure files. In either case, compressed files and archives in Z-, GZ-, or BZ2-format are also supported. The user can add further files, delete redundant ones, and decompose structure files into all their models and chains. The user may select a subset of chains to be compared against each other, or perform an all-against-all chain comparison. If a PDB file was considered invalid, e.g. due to incomplete or ambiguous data within the file, the user has the opportunity to correct the errors before submitting the request for calculation. That is, the *Dataset Management *provides a flexible and user friendly interface for preparing the dataset for the further comparison in just a few steps.

### Calculation Management

**Figure 2:**
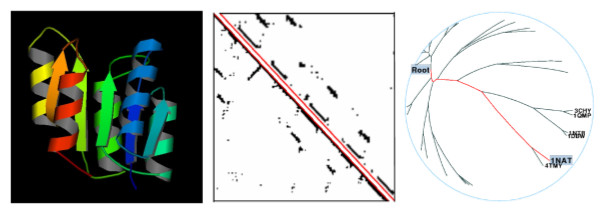
**Building the Protein Universe**. 3D proteins structures (left, [68]) can be represented as 2D *contact maps *that are used to compare pairs of proteins (centre) according to their USM and MaxCMO *similarities*. The resulting *similarity matrices *can be clustered in order to produce a hierarchical tree (right, [51]). *Spoof *protein from *Bacillus Subtilis *(1NAT) from the Skolnick dataset [23].

Once the protein files have been validated, the user must specify the calculation parameters, including the similarity methods to be used. Each comparison method requires specific parameters, which the *Calculation Management *allows to be set up. In the case of a similarity calculation with the USM method a *USM equation *[24,59] and a *compression type *must be chosen; for the MaxCMO method the *number of restarts *for the randomised solver has to be specified. The more restarts, the better the overlap values obtained, but this, of course, comes at the cost of compute time. All other methods take their standard parameters. When using the USM or MaxCMO method, each protein structure is then automatically converted into a contact map (Figure 2 – centre), based on a user-defined *distance threshold *and an *exclusion window*. The latter parameter specifies the number of nearest neighbour atoms in the sequence to be ignored while calculating the contact map.

As there is no general agreement on how to best represent a protein structure for comparison purposes, either *C*_*α *_atoms [60-62] or *C*_*β *_atoms [63-66] can be chosen. The former representation focuses on the structure's backbone, whereas the latter one takes the residues' side chains into account. Alternatively and in contrast to other web servers (e.g. [67]), ProCKSI offers to calculate the residues' centres of mass in order to include both the backbone and side chain contributions at once.

### Results Management

After a similarity comparison request has been submitted, it is added to a queue and the server returns a confirmation page that directly links to the *Overview Manager*. This gives a summary of all calculation parameters and the status, the submission, start and end times of all methods (tasks) that have been requested. As soon as all tasks have finished, the user receives a notification email and the expiration time (currently 7 days) for the entire request. Thereafter, the data are deleted.

ProCKSI returns a large variety of data and intermediate results that are handled through the *Structures*, *Task *and *Analysis Management *subsystems. These are described next.

#### Structure Management

As the USM and MaxCMO calculations both require contact maps as input, these are prepared before the actual similarity calculations take place. For each protein, all partial results are accessible for download and include a list of selected atoms, the protein's distance matrix and contact map, files with absolute and relative contacts and its contact vector (contact numbers). Thumbnails for the contact map of each protein are generated automatically, whereas high-quality pictures in different formats (PNG, PS, EPS), user-defined sizes and colours can be produced on demand. Two versions of contact map representations are available: dot matrix, and vertex & edges. Additionally, the input protein structure can be displayed as plain text or as a static image preview [68]; an interactive *VRML *representation in 3D is also available and allows the user to explore and analyse the protein further (rotation, zoom, etc). The secondary structure is displayed as given in the PDB file (Figure 2 – left). For further and more detailed information about a protein, a direct link to the corresponding pages at the PDB repository [52], the SCOP [53,54] and CATH [55,56] structures classification databases, and the iHOP cross-literature database [57,58] are also given.

#### Task Management

As a request will usually involve several similarity methods, each of these is assigned to a separate *Task Manager*, which gives an overview of all similarity measures produced by any similarity method. These are Z-scores when using DaliLite or CE, overlap values in case of MaxCMO, whereas TM-align and FAST produce TM-scores and SN-scores, respectively. Most methods provide RMSD values and the number of aligned residues, too. The USM method only returns the USM-score. Additionally, the user can access the natural output of the corresponding similarity method, e.g. the structural alignments or a list of structurally equivalent residue ranges.

#### Analysis Management

Once a task has finished, its similarity measures are available through the *Analysis Management *where they can be visualised and analysed. As the different similarity matrices are often either sensitive to protein size or do not have a fixed range of values, they must be normalised so that they can be used later to calculate a *consensus *similarity. Hence, besides providing the original similarity matrices (SM), ProCKSI also converts these into a standardised similarity matrix (SSM): each entry in the SSM matrix lies in the range [0, 1], with 0 describing the best (i.e. most similar), and 1 the worst (i.e. most dissimilar) similarity between two structures within the given set of proteins. The SSM matrix is then taken as input for clustering the protein set with one of a variety of hierarchical clustering methods, including e.g. the *Unweighted Pair Group Method with Arithmetic mean *(UPGMA) [69] or the *Ward's Minimum Variance *(WMV) method [70]. ProCKSI uses a local version of the *Clustering Calculator *[71] that outputs a plain text representation of the hierarchical tree and a file in the *PHYLIP*-format [72]. Especially useful for visualising large data sets, ProCKSI generates a *HyperTree *view [51] that opens as a Java applet from within the browser. This allows the user to display the protein set hierarchies as a circular, linear or as a hyperbolic tree. One benefit of using the hyperbolic representation is that it facilitates the navigation through the entire tree with a "fish-eye" perspective allowing to zoom in/out of regions of interest (Figure 2 – right). Not only can individual similarity measures be analysed as described before, but also the SSM can be combined in order to give a *consensus *picture of similarity. In turn, this consensus similarity matrix can be used as input for the clustering process thus obtaining a consensus hierarchical clustering tree.

## Results

ProCKSI's core technologies, namely the USM and MaxCMO methods, and external servers and methods, namely the DaliLite, CE, FAST, and TM-align methods, have been introduced and evaluated independently in the past [8,11,20,22-24,27,28,49,50]. In this section though, we concentrate on ascertaining the *added value *of having, on the one hand, a unique interface to access all the previously mentioned methods, and on the other hand, the facility both to compare similarity assessments and to compute a consensus measure. We will present several case studies focusing on different aspects of ProCKSI's features and performance: a) the evaluation of some recent CASP results, introducing ProCKSI's new *Consensus *method, b) a new study reproducing the Hanks' and Hunt's classification scheme of protein kinases [73], which was originally derived from sequence comparisons, but this time verifying it using a consensus of different structural similarity methods, and c) an analysis of all methods implemented in ProCKSI using Receiver Operator Characteristics (ROC) with the Rost and Sander dataset. In addition to an analysis of the influence of different similarity methods and measures on the *quality *of the consensus, we conclude with benchmark tests measuring ProCKSI's total *time *needed to produce this similarity consensus.

### Evaluation of the CASP6 Results

The evaluation of the CASP6 results is a challenging task, as it involves many protein structure files of a widely varying degree of similarity. Although protein scientists will often be interested in good alignments between pairs of *closely *related proteins, the capability of properly aligning *distantly *related structures is useful in ab-initio (new fold) structure prediction and the assessment of the predicted structures [74]. In CASP, the evaluators need to deal with literally tens of thousands of protein structure candidates that are often not too similar to the targets. The case of very different protein structures sometimes baffles structural alignment methods including LGA [12] that are regularly used to evaluate CASP results. ProCKSI, on the other hand, is likely to be less prone to producing a misleading ranking, because it harvests the results from various methods averaging them in order to produce a consensus.

For our experiments, we have chosen targets from both the CASP6 *CM/easy *and *CM/hard *category. The differentiation of targets into *easy *and *hard *is related to the degree of difficulty of predicting a model for the target using a given template, but does not reflect the evaluation process. For *CM/easy *targets, homologous structures can be found in databases, which might lead to many fairly good models. These can be very similar to each other and difficult to rank properly. On the other hand, the evaluation of the *CM/hard *category is not straightforward either, as the structural similarity between model and target can be very low, due to the more difficult structural prediction task.

In the following, we discuss three examples (targets T0231, T0211 and T0196) that illustrate how ProCKSI deals with the difficulties described above. The targets were compared to all models proposed by the prediction servers that produced a native-like protein model including side chains. We used C_*β *_atoms to represent the protein structure in this experiment in order to be consistent with the assessment of the CASP6 experiment [66] thus taking the positions of the side chains into account. ProCKSI's results from methods using contact maps, namely USM similarity, MaxCMO/Overlap and MaxCMO/Align values, and its new *ProCKSI/Consensus *method (using a total-evidence approach [47] combining all three taking the arithmetic average) are compared against GDT-TS, the main measure for structural similarity in CASP [75]. We provide the GDT-TS ranking results obtained from a *sequence dependent analysis *(SDA) as used in the official CASP evaluation procedure, and additionally from a *sequence independent analysis *(SIA), as MaxCMO's algorithm works in this mode.

Table 1 shows the ranking results of target T0231, a protein from the *CM/easy *category. This structure is the most conserved structure in CASP6 [76], i.e. homologous ones can be found in various databases, making its prediction simpler than it would otherwise be. The availability of several good, only slightly different models, makes the ranking process difficult as small differences between the candidate structures must be detected and evaluated. ProCKSI's results are in very good agreement with GDT-TS (SDA), the community's gold standard. At least three targets ranked in the top five places by GDT-TS (SDA) can be found in similar places when using *any *of ProCKSI's similarity measures. More specifically, not only does ProCKSI find some major agreement in the *most similar *models for this target but also the three *least similar *models as ranked by MaxCMO/Overlap, MaxCMO/Align and ProCKSI/Consensus match perfectly the ranking of GDT-TS (SDA).

**Table 1:** Evaluation of CASP Target T0231. Comparison of the ranking results of target T0231 against 24 server predicted models, using different similarity methods. The GDT-TS results are obtained from calculations with *sequence independent analysis *(SIA) and *sequence dependent analysis *(SDA)

Ranking	GDT-TS SDA	GDT-TS SIA	USM	MaxCMO Overlap	MaxCMO Align	ProCKSI Consensus
1	TS030	TS289	TS338	TS030	TS338	TS338
2	TS207	TS519	TS186	TS139	TS207	TS030
3	TS242	TS283	TS030	TS338	TS242	TS207
4	TS186	TS139	TS207	TS207	TS033	TS186
5	TS338	TS324	TS400	TS242	TS139	TS242

20	TS451	TS400	TS451	TS451	TS114	TS451
21	TS101	TS114	TS381	TS114	TS381	TS381
22	TS304	TS101	TS019	TS304	TS304	TS304
23	TS519	TS019	TS304	TS519	TS519	TS519
24	TS019	TS304	TS519	TS019	TS019	TS019

Focusing on target T0211 of the *CM/hard *category [76] we find that GDT-TS (SDA) and ProCKSI/Consensus consider the same models for the first and second best models and that also the last six places, i.e. worst models, show considerable agreement (Table 2).

**Table 2:** Evaluation of CASP Target T0211. Comparison of the ranking results of target T0211 against 24 server predicted models, using different similarity methods. The GDT-TS results are obtained from calculations with *sequence independent analysis *(SIA) and *sequence dependent analysis *(SDA)

Ranking	GDT-TS SDA	GDT-TS SIA	USM	MaxCMO Overlap	MaxCMO Align	ProCKSI Consensus
1	TS451	TS213	TS451	TS451	TS451	TS451
2	TS283	TS381	TS263	TS283	TS289	TS283
3	TS101	TS324	TS101	TS400	TS283	TS400
4	TS207	TS290	TS324	TS381	TS381	TS381
5	TS375	TS263	TS207	TS324	TS324	TS324

20	TS338	TS019	TS114	TS519	TS304	TS519
21	TS352	TS033	TS352	TS186	TS338	TS338
22	TS186	TS352	TS033	TS338	TS033	TS033
23	TS304	TS186	TS186	TS033	TS352	TS352
24	TS519	TS519	TS338	TS352	TS186	TS186

Interestingly, the results obtained by ProCKSI and GDT-TS running in sequence independent mode differ more, when given targets T0231 and T0211, than when GDT-TS operates in sequence dependent mode. For example, considering T0231, we obtain no agreement between ProCKSI's top 5 models and GDT-TS (SIA) and only two (ProCKSI/Consensus) or three (MaxCMO) matches in the bottom 5 ranked models. These are quite surprising results as one would have expected the results of ProCKSI to match the sequence independent operation mode of GDT-TS better than the sequence dependent one, as ProCKSI does not use sequence information. Noting that CASP results are evaluated on the sequence dependent mode and that ProCKSI produced good agreement with it, we will investigate in the near future whether adding GDT-TS (SIA) to ProCKSI's pool of methods would be advantageous.

**Figure 3:**
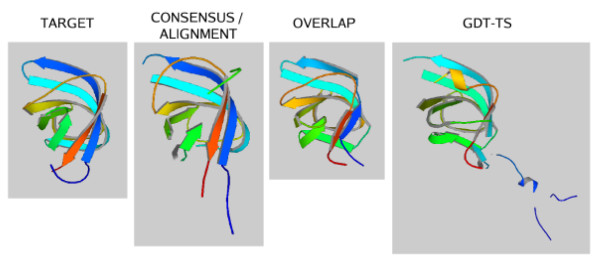
**Evaluation of Models against CASP Target T0196**. CASP target T0196 was compared against all submitted server models using ProCKSI's similarity methods USM, MaxCMO, and its new Consensus method. The most similar structures to the target detected by each method are displayed next to each other. – The protein structure pictures were generated by ProCKSI using MolScript [68].

Using target T0196 as a third example taken from the *CM/hard *category, we show that the combination of similarity criteria into a consensus can sometimes detect a better model than GDT-TS (SDA)(see Table 3 for details). Figure 3 illustrates the 3D structures of those models that are considered by the different methods to be the most similar to the target structure, called the "winner" of a certain method in the following. The ProCKSI/Consensus similarity measure detects a model that better resembles the overall structural features of T0196. More specifically, the candidate model selected by GDT-TS (SDA) has a fairly long segment of the chain that was not predicted correctly (blue), a helix structure was incorrectly suggested (orange) and the *β *sheets are in the wrong places (green). This model is ranked last by ProCKSI/Consensus.

**Table 3:** Evaluation of CASP Target T0196. Comparison of the ranking results of target T0196 against 22 server predicted models, using different similarity methods. GDT-TS results are obtained from calculations with *sequence independent analysis *(SIA) and *sequence dependent analysis *(SDA)

Ranking	GDT-TS SDA	GDT-TS SIA	USM	MaxCMO Overlap	MaxCMO Align	ProCKSI Consensus
1	TS223	TS352	TS352	TS030	TS352	TS352
2	TS213	TS290	TS030	TS381	TS030	TS030
3	TS451	TS381	TS400	TS352	TS381	TS381
4	TS033	TS030	TS030	TS400	TS290	TS290
5	TS376	TS223	TS400	TS304	TS324	TS324

18	TS338	TS338	TS289	TS338	TS304	TS338
19	TS324	TS242	TS376	TS283	TS113	TS113
20	TS304	TS033	TS113	TS113	TS283	TS283
21	TS289	TS207	TS213	TS213	TS213	TS213
22	TS352	TS375	TS223	TS223	TS223	TS223

Next, we analysed the sequence similarities between the target structure and the winner of each method. Using the MaxCMO method to produce all sequence alignments, we obtained up to 76.7% (target vs. winner of GDT-TS), up to 98.6% (target vs. winner of MaxCMO/Overlap) and up to 95.8% (target vs. winner of USM and ProCKSI/Consensus) correctly aligned residues, having taken the best results of multiple MaxCMO runs. This illustrates that the MaxCMO method does not only detect the most similar model according to its overlap values, but also gives the better alignment with the highest sequence similarity. When using GDT-TS in sequence independent mode instead, both methods suggest the same model for the best structural match and even agree with almost all models within the top five in the ranking.

Summing up, we found a very good agreement between ProCKSI/Consensus and CASP's GDT-TS method, although the two run in different modes: the former obtains its results from *sequence-independent *calculations while the latter additionally uses sequence information. When both methods suggest a different winner in their rankings, the ProCKSI/Consensus method can detect a better model with a higher similarity (value) and even a higher sequence similarity.

### Structural Comparison and Clustering of Protein Kinases

In the following, we perform an additional (and more detailed) analysis of ProCKSI's functionality by concentrating on a set of protein kinases (PK). These are then compared and clustered according to their structural similarity. These structure-only based results are then compared with the original classification scheme by Hanks and Hunter (HH) [73] that was based on sequence similarity. As was the case for the CASP targets, ProCKSI's integration of several structural similarity criteria allows it to reproduce the original classification without using sequence information.

Kinases are proteins that catalyse the transfer of a phosphate to a protein substrate and form a reversible equilibrium with phosphatases as their counterpart [77]. They comprise a huge group of enzymes that play an essential role in most of the major cellular processes such as cellular differentiation and repair, cell proliferation, etc [78]. In an attempt to organise the set of protein kinases, Hanks and Hunter [73] classified them accordingly to their sequence into 5 broad groups (super-families), 44 families, and 51 domains (sub-families). Several additions and refinements to this classification (e.g. [79-82]) were later introduced.

#### Dataset Preparation

As the dataset for our experiments, we have chosen the structures published on a mirror site [83] of the *Protein Kinase Resource *(PKR) web site [77,78,84], which is an online compendium for information on protein kinases [77]. We use Hanks' and Hunter's original classification as it goes hand in hand with this dataset that comprises 46 structures from 9 different groups (super-families), namely 1) *cAMP Dependent Kinases*, 2) *Protein Kinases C*, 3) *Phosphorylase Kinases*, 4) *Calmodulin Kinases*, 5) *Casein Kinases*, 6) *Cyclin Dependent Kinases*, 7) *Tyrosine Kinases*, 8) *Mitogen Activated Kinases*, and 9) *Twitchen Kinases*. For each protein, we obtained detailed information about its class, fold, superfamily, family, protein, and species from the SCOP database, release 1.69 [85], which is summarised in Table 4. It should be mentioned that Hanks' and Hunter's original classification scheme showed separate super-families for *Tyrosine Kinases *(TK) and *Serine/Threonin Kinases *(S/TK). Starting with SCOP release 1.65, these have been combined into one single family comprising all protein kinases with a characteristic catalytic subunit [54]. In this text, we refer to the new classification, but for the sake of completeness, the old classification is denoted in parentheses.

**Table 4:** Protein Kinase Dataset. Detailed SCOP classification for each protein domain in the Protein Kinase (PK) dataset, grouped according to Hanks' and Hunter's (HH) original classification scheme

**HH Cluster**	**Sub Cluster**	**Protein Domain**	**SCOP Classification Level**					
			**Class**	**Fold**	**Superfamily**	**Family**	**Domain**	**Species**
1	A	d1apme_	*α *+ *β*	PK-like	PK-like	PK c.s. (S/TK)	cAMP-dep. PK, c.s.	mouse
		d1atpe_	*α *+ *β*	PK-like	PK-like	PK c.s. (S/TK)	cAMP-dep. PK, c.s.	mouse
		d1bkxa_	*α *+ *β*	PK-like	PK-like	PK c.s. (S/TK)	cAMP-dep. PK, c.s.	mouse
		d1fmoe_	*α *+ *β*	PK-like	PK-like	PK c.s. (S/TK)	cAMP-dep. PK, c.s.	mouse
		d2cpke_	*α *+ *β*	PK-like	PK-like	PK c.s. (S/TK)	cAMP-dep. PK, c.s.	mouse
	
	B	d1cdka_	*α *+ *β*	PK-like	PK-like	PK c.s. (S/TK)	cAMP-dep. PK, c.s.	pig
		d1cmke_	*α *+ *β*	PK-like	PK-like	PK c.s. (S/TK)	cAMP-dep. PK, c.s.	pig
		d1ctpe_	*α *+ *β*	PK-like	PK-like	PK c.s. (S/TK)	cAMP-dep. PK, c.s.	pig
	
	C	d1stce_	*α *+ *β*	PK-like	PK-like	PK c.s. (S/TK)	cAMP-dep. PK, c.s.	cow
		d1ydre_	*α *+ *β*	PK-like	PK-like	PK c.s. (S/TK)	cAMP-dep. PK, c.s.	cow
		d1ydse_	*α *+ *β*	PK-like	PK-like	PK c.s. (S/TK)	cAMP-dep. PK, c.s.	cow
		d1ydte_	*α *+ *β*	PK-like	PK-like	PK c.s. (S/TK)	cAMP-dep. PK, c.s.	cow

2	-	d1ptq_	small	PK c.-r. domain	PK c.-r. domain	PK c.-r. domain	PK C-delta (PKCdelta)	mouse
		d1ptr_	small	PK c.-r. domain	PK c.-r. domain	PK c.-r. domain	PK C-delta (PKCdelta)	mouse

3	-	d1phk_	*α *+ *β*	PK-like	PK-like	PK c.s. (S/TK)	γ-subunit glycogen Phk	rabbit

4	A	d1a06_ _	*α *+ *β*	PK-like	PK-like	PK c.s. (S/TK)	Calmodulin-dep. PK	rat
	
	B	d1cdma_	*α*	EF Hand-like	EF-hand	Calmodulin-like	Calmodulin	cow
		d1cm1a_	*α*	EF Hand-like	EF-hand	Calmodulin-like	Calmodulin	cow
		d1cm4a_	*α*	EF Hand-like	EF-hand	Calmodulin-like	Calmodulin	cow

5	A	d1lr4a_	*α *+ *β*	PK-like	PK-like	PK c.s. (S/TK)	Casein kinase-2, CK2	maize
	
	B	d1csn_	*α *+ *β*	PK-like	PK-like	PK c.s. (S/TK)	Casein kinase-1, CK1	fission yeast
		d2csn_	*α *+ *β*	PK-like	PK-like	PK c.s. (S/TK)	Casein kinase-1, CK1	fission yeast

6	-	d1aq1_	*α *+ *β*	PK-like	PK-like	PK c.s. (S/TK)	Cyclin-dep. PK, CDK2	human
		d1fina_	*α *+ *β*	PK-like	PK-like	PK c.s. (S/TK)	Cyclin-dep. PK, CDK2	human
		d1hck_	*α *+ *β*	PK-like	PK-like	PK c.s. (S/TK)	Cyclin-dep. PK, CDK2	human
		d1hcl_	*α *+ *β*	PK-like	PK-like	PK c.s. (S/TK)	Cyclin-dep. PK, CDK2	human
		d1jsua_	*α *+ *β*	PK-like	PK-like	PK c.s. (S/TK)	Cyclin-dep. PK, CDK2	human

7	A	d1ad5a1	β	SH3-like barrel	SH3-domain	SH3-domain	Hemapoetic cell kinase Hck	human
		d1ad5a2	*α *+ *β*	SH2-like	SH2 domain	SH2 domain	Hemopoetic cell kinase Hck	human
		d1ad5a3	*α *+ *β*	PK-like	PK-like	PK c.s. (TK)	Hemopoetic cell kinase Hck	human
		d1fmk_ 1	β	SH3-like barrel	SH3-domain	SH3-domain	c-src protein TK	human
		d1fmk_ 2	*α *+ *β*	SH2-like	SH2 domain	SH2 domain	c-src TK	human
		d1fmk _3	*α *+ *β*	PK-like	PK-like	PK c.s. (TK)	c-src TK	human
		d2hcka_1	β	SH3-like barrel	SH3-domain	SH3-domain	Hemapoetic cell kinase Hck	human
		d2hcka_2	*α *+ *β*	SH2-like	SH2 domain	SH2 domain	Hemopoetic cell kinase Hck	human
		d2hcka_3	*α *+ *β*	PK-like	PK-like	PK c.s. (TK)	Haemopoetic cell kinase Hck	human
		d2ptk _1	β	SH3-like barrel	SH3-domain	SH3-domain	c-src protein TK	chicken
		d2ptk_ 2	*α *+ *β*	SH2-like	SH2 domain	SH2 domain	c-src TK	chicken
		d2ptk _3	*α *+ *β*	PK-like	PK-like	PK c.s. (TK)	c-src TK	chicken
	
	B	d1aotf_	*α *+ *β*	SH2-like	SH2 domain	SH2 domain	TK Fyn	human
		d1blj_	*α *+ *β*	SH2-like	SH2 domain	SH2 domain	P55 Blk protein TK	mouse
		d1csya_	*α *+ *β*	SH2-like	SH2 domain	SH2 domain	Syk TK	human
		d1cwea_	*α *+ *β*	SH2-like	SH2 domain	SH2 domain	p56-lck TK	human
	
	C	d1fgka_	*α *+ *β*	PK-like	PK-like	PK c.s. (TK)	Fibroblast growth factor receptor 1	human
		d1ir3a_	*α *+ *β*	PK-like	PK-like	PK c.s. (TK)	Insulin receptor	human
		d1irk_	*α *+ *β*	PK-like	PK-like	PK c.s. (TK)	Insulin receptor	human
		d3lck_	*α *+ *β*	PK-like	PK-like	PK c.s. (TK)	Lymphocyte kinase (lck)	human

8	A	d1erk_	*α *+ *β*	PK-like	PK-like	PK c.s. (S/TK)	MAP kinase Erk2	rat
	
	B	d1ian_ _	*α *+ *β*	PK-like	PK-like	PK c.s. (S/TK)	MAP kinase p38	human
		d1p38__	*α *+ *β*	PK-like	PK-like	PK c.s. (S/TK)	MAP kinase p38	mouse
		d1wfc_	*α *+ *β*	PK-like	PK-like	PK c.s. (S/TK)	MAP kinase p38	human

9	A	d1koa _1	β	IG-like β-sandwich	IG	I set domains	Twitchin	nematode
		d1ko_a 2	*α *+ *β*	PK-like	PK-like	PK c.s. (S/TK)	Twitchin, kinase domain	Caenorhabditis elegans
	
	B	d1kob_a	*α *+ *β*	PK-like	PK-like	PK c.s. (S/TK)	Twitchin, kinase domain	California sea hare

IG = Immunoglobulin, PhK = Tyrosine Phosphorylase Kinase, S/TK = Serine/Threonin Kinase, TK = Tyrosine Kinase, c.s. = catalytic subunit, c.-r. = cysteine-rich., dep. = dependent

A further first analysis of the dataset revealed that protein 1RGS, a double stranded *β*-helix, was given as the only *all beta *protein within an *alpha+beta *class (HH cluster 1), and was therefore removed from the dataset. A further, more detailed analysis of the remaining 45 structures revealed a more fine-grained similarity structure than that suggested by Hanks and Hunter. In most of the clusters given in Table 4, the protein kinases can be sub-divided into sub-clusters with common features. This could be either a common class (e.g. cluster 4), a common fold (e.g. cluster 7), or even a common species (e.g. cluster 1). The members of clusters 2, 3 and 6 cannot be further differentiated as they share the same features up to the species level, respectively. In order to capture this intrinsic similarity structure, we have sub-divided the HH clusters according to the biggest set of common features, labelling these with letters in addition to the original cluster number. Clusters 2, 3 and 6 are not considered as they cannot be further sub-divided. To illustrate this, consider proteins 1AD5 and 1FGK, both belonging to HH cluster 7 (Tyrosine Kinases). The former is built up from multiple domains while the latter has just one domain. These are therefore put into two different sub-clusters, 7A and 7C.

#### Kinase Structural Classification Results

From the available methods in ProCKSI (USM, MaxCMO, DaliLite, CE, TM-align, FAST), we have performed similarity calculations between all the 45 Kinases using the USM, the MaxCMO and the DaliLite methods. These 45 proteins imply at least 1035 pairwise comparisons per parameter setting of each algorithm. These include the comparison of a structure with itself, as the self-similarity values are needed to standardise the final similarity matrix. On top of each request using one of three different contact map thresholds, seven different clustering methods can be applied. ProCKSI's interface handles the set up of all these comparisons in an automatic and user-friendly way.

In particular, C_*α *_atoms were chosen to represent the protein structures with all methods, taking into account that DaliLite uses them by default. Distance thresholds of 5.0 Å, 7.5 Å and 10.0 Å were chosen in order to produce the contact maps necessary for USM and MaxCMO. Rather than prescribing a given clustering method, following ProCKSI's philosophy, our decision support system allows the user to choose, and seamlessly try, different clustering algorithms on his/her datasets. In this example the similarity matrices obtained from USM, MaxCMO and DaliLite were standardised and fed into each of the clustering methods available in ProCKSI. For brevity we report only the results using the 7.5Å and the WMV clustering algorithm.

**Figure 4:**
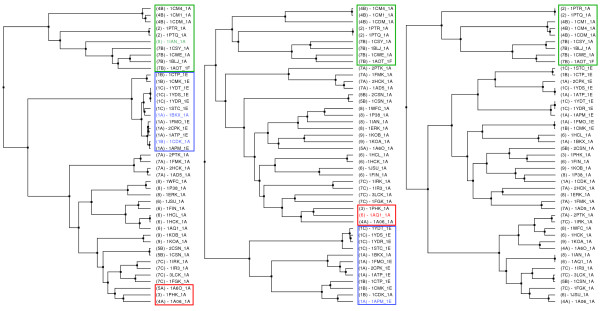
**Clustering the Kinase Dataset using Single Similarity Measures**. The Kinase dataset was clustered with the Ward's Minimum Variance (WMV) method in conjunction with the MaxCMO/Overlap (left), the USM (middle), and the DaliLite/Z (right) similarity measures. The meaning of the different coloured boxes is explained in the text in detail. – The hierarchical tree images were generated by ProCKSI using HyperTree [51].

**Figure 5:**
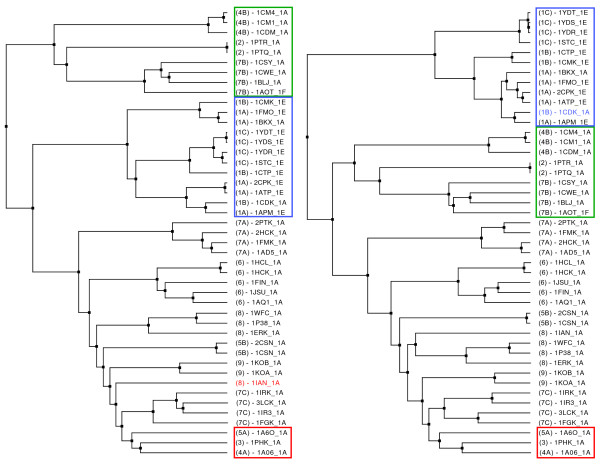
**Clustering the Kinase Dataset using Consensus Similarity Measures**. The Kinase dataset was clustered with the Ward's Minimum Variance (WMV) method in conjunction with a consensus similarity measure of the USM and DaliLite/Z (left), and the USM, DaliLite/Z, and MaxCMO/Overlap (right) similarity measures. The meaning of the different coloured boxes is explained in the text in detail. – The hierarchical tree images were generated by ProCKSI using HyperTree [51].

The MaxCMO method distinguishes the kinases in our dataset clearly upon the class/fold level and separates them into two clusters (Figure 4 – left): *alpha+beta*/PK-like proteins and others. The latter comprise all small proteins (2), *all alpha *proteins with EF Hand-like fold (4B), and such from the *alpha+beta *class but with SH2-like fold (7B), all of them being identified and clustered correctly. One could assume that multidomain proteins with diverse classes/folds (7A; 1KOA) should be found in this cluster, too, but a more in-depth analysis revealed that the domains of these proteins show mainly *alpha+beta*/PK-like fold (>62.5% in the entire structure). Consequently, they are correctly grouped together with proteins resembling the same class/fold properties. As this cluster is detected by each similarity measure almost always correctly, it is highlighted with a green box in all dendrograms (Figures 4 and 5). While DaliLite wrongly adds a fairly different protein to this cluster (1IAN from HH cluster 8), the USM method just reverses the order of the penultimate and the last clustering step. In addition, both the USM and DaliLite/Z measures are able to detect similarities up to the species level (Figure 4 – middle). Consider, for instance, HH cluster 1 (blue box) containing similar kinases from mice, pigs and cows, which are clustered comparably well (errors indicated in blue within the blue cluster). Both methods also produce a "mixed bag" of proteins (red box) that are less similar to all others outside the green cluster, with the USM method misplacing 1AQ1 (6) into the red cluster.

The results illustrate both the strengths and minor flaws of different similarity methods taken as independent criteria of similarity. We proceed next to analyse the results of the Kinase structural classification when taking these criteria in combination.

The previous analysis shows that including similarity matrices derived from alignment numbers (MaxCMO/Align, DaliLite/Align) always cluster proteins within the green box correctly, but partially destroy the good clustering of the other proteins. Thus, they are not considered as candidates for producing a high-quality *consensus *clustering for this dataset. The best result was obtained combining the USM and DaliLite/Z similarity measures, which reproduced the red, blue and green clusters correctly (Figure 5 – left). Both the DaliLite's error within the green cluster and the USM error within the red cluster were corrected while the proteins in the blue cluster were correctly classified. Surprisingly enough, adding the MaxCMO/Overlap similarity measure, which was only able to produce the correct clustering within the green cluster, still gives a comparably good result (Figure 5 – right).

Taken together, these results show that the combination of a range of algorithms that employ different similarity criteria has the potential to overcome the inherent weaknesses in each one of them, and thus is able to produce a robust more similarity result.

### Evaluation of Multiple Similarity Comparison Methods using ROC Curves

In the previous section, we investigated in detail by manual inspection how the similarity comparison methods USM, MaxCMO and DaliLite can be combined in order to achieve an optimal consensus result. In this section, we take the next step towards a fully-automated decision support system by analysing the quality and performance of the six different similarity comparison methods currently included in ProCKSI by means of *Receiver Operator Characteristics *(ROC) [86]. These are USM, MaxCMO, DaliLite, CE, TM-align, and FAST, providing a total number of 15 similarity measures (compare section *Task Management *for details).

In the following, we describe the experimental setup, explain how ROC curves are generated, and employ this technique to determine the most promising methods to include in order to produce an even better consensus method.

#### Dataset and Gold Standard

For our analysis, we have chosen the Rost and Sander dataset (RS126), which was designed for the secondary structure prediction of proteins with a pairwise sequence similarity of less than 25% [87]. Here, we not only compare the proteins' secondary structures, but analyse the performance of ProCKSI's similarity comparison methods according to the proteins' classification as given by SCOP, release 1.69 [85]. We adopted this manually curated database as our gold standard containing expert knowledge for each of its hierarchical classification levels: *Class*, *Fold*, *Superfamily*, *Family*, *Protein*, and *Species*.

The dataset itself consists of 126 globular proteins, 18 of them with more than one domain. In order to allow the comparison of entire chains instead of breaking down the protein into domains, we have merged and re-classified all of a protein's multiple domains. In contrast to other over-simplified approaches like [88], where all multi-domain proteins were merged with SCOP's already existing "multi-domain" class, we tried to preserve as much information as possible on each hierarchy level. If two domains disagreed in all classification levels, we also merged and re-classified them as "multi-domain". Moreover, moving down the hierarchy from the *Class *to the *Species *level, we kept the original classification for those levels that matched. For instance, if the class of two domains was given as "all alpha", but they showed different classifications for all other levels, then the class of the entire chain was kept as "all alpha", but all other levels were re-classified as "multi-domain". Thus, in contrast to the approach of [88], comparing this chain with another "all alpha" chain counts as a correct classification (true positive) in the ROC analysis (see below).

#### Introduction to ROC Analysis

ROC analyses have been widely employed, e.g. in signal detection theory [89], machine learning [90], and diagnostic testing in medicine [91]. Recently, they have also been used for the evaluation of structural similarity and alignment methods [88,92,93].

The performance of such a comparison or alignment method is measured by its ability to *predict *the degree of similarity between pairs of proteins, and to produce a relative ranking of similar (positive) and dissimilar (negative) pairs. The fraction of correctly classified positives (true positives) and the number of wrongly classified positives (false positives) in relation to the real number of positives gives the *true positive rate *(TPr) and *false positive rate *(FPr), respectively. In a ROC graph, TPr and FPr are plotted against each other using a continuously varying decision threshold discriminating between true and false positives. The diagonal line between (0,0) and (1,1) denotes classifiers without any predictive power as they produce the correct classification just by chance [88]. The further a ROC curve is to the north-west in the graph, the better is the classifier, whereas classifiers in the south-east region have strong predictive power, but lead to wrong (opposite) conclusions [86].

In order to compare the complex performance of different similarity comparison methods, a ROC curve can be reduced to a single, scalar measure given as the *Area Under the Curve *(AUC). As the best method will have the uppermost (north-western) curve, it will have the largest AUC value, whereas an AUC value below 0.5 indicates an incorrect prediction.

#### Comparison of Methods using ROC curves

**Figure 6:**
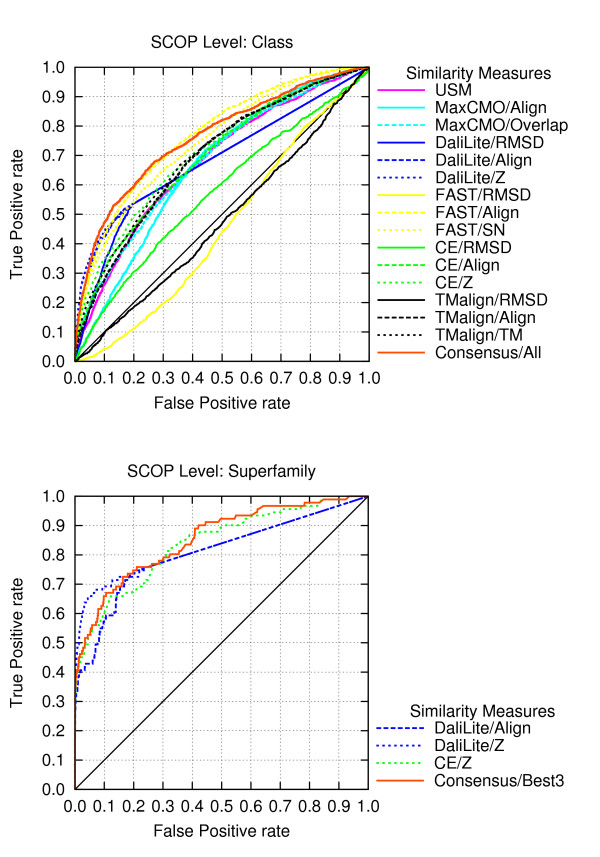
**ROC Analysis for the Rost and Sander Dataset**. ROC analysis for all available similarity comparison methods in ProCKSI for the Rost and Sander dataset using SCOP's *Class *level as gold standard (top). ROC analysis showing the better performance of ProCKSI's *Consensus/Best3 *method compared to each contributing single one using SCOP's *Superfamily *level as gold standard (bottom).

For each classification level, we generated ROC graphs using all available similarity measures, and combined all of them in order to produce a consensus (Consensus/All). Figure 6 shows the ROC graph for the *Class *level as a representative in order to explain some details of the graphs further: In this graph, all DaliLite measures display an unusual straight line from an FP rate of about 0.2 onwards. This artifact emerges from the fact that DaliLite does not return any similarity values for pairs of proteins with very low similarity. As a consequence, all those pairs are assigned the worst possible value (1.0) in the standardised similarity matrix and cannot be ranked unambiguously for the generation of the ROC curves. Not having more information at hand to predict the method's expected performance, we do not calculate any ROC points for any but the first of subsequent pairs with the same similarity value [86]. We obtain a straight line between the latter and the (1,1) point, which is always present.

In the following, we analyse the performance of all included similarity measures using AUC values (Table 5). We found consistently in all hierarchy levels that RMSD values do not seem to be good similarity measures, giving very low AUC values, even below 0.5. On the other hand, it is DaliLite/RMSD that could be used as a good similarity predictor ranking within the best four methods in almost all hierarchy levels except the *Class *level. Besides, the latter is the only level, where both FAST/Align and FAST/SN rank within the best three methods. In all other levels, CE/Z, DaliLite/Z, and DaliLite/Align show the best performance.

**Table 5:** ROC Analysis for the Rost and Sander Dataset. Analysis of the performance of different similarity measures in terms of AUC values for each SCOP classification level using the Rost and Sander dataset. The Consensus measures are composed of ^*a *^CE/Z, DaliLite/Z, FAST/Align, MaxCMO/Overlap, TM-align/TM, and USM/USM for the Class and Species level, and CE/Z, DaliLite/Z, FAST/SN, MaxCMO/Overlap, TM-align/TM, and USM/USM for all remaining levels, ^*b *^FAST/Align, FAST/SN, and DaliLite/Z for the Class level, and CE/Z, Dali/Z, and Dali/Align for all remaining levels, and ^*c *^FAST/Align and FAST/SN for the Class level, and CE/Z and Dali/Z for all remaining levels.

Method	Measure	AUC Values for SCOP Classification Level					
		Class	Fold	Superfamily	Family	Protein	Species
CE	RMSD	0.574	0.776	0.754	0.718	0.625	0.626
	Align	0.694	0.704	0.660	0.643	0.503	0.486
	Z	0.712	0.848	0.838	0.826	0.769	0.759

DaliLite	RMSD	0.677	0.807	0.794	0.786	0.746	0.751
	Align	0.693	0.827	0.807	0.792	0.755	0.759
	Z	0.696	0.846	0.830	0.817	0.792	0.797

FAST	RMSD	0.454	0.530	0.514	0.490	0.322	0.303
	Align	0.770	0.800	0.773	0.757	0.684	0.672
	SN	0.747	0.802	0.779	0.761	0.684	0.671

MaxCMO	Align	0.665	0.685	0.687	0.730	0.672	0.667
	Overlap	0.682	0.751	0.743	0.769	0.706	0.693

TM-align	RMSD	0.475	0.624	0.602	0.550	0.354	0.336
	Align	0.695	0.747	0.733	0.741	0.656	0.645
	TM	0.705	0.773	0.756	0.751	0.673	0.666

USM	USM	0.678	0.686	0.680	0.683	0.578	0.566

Consensus	All	0.764	0.816	0.797	0.793	0.724	0.712
	BestOfEach^*a*^	0.759	0.818	0.804	0.803	0.746	0.740
	Best3^*b*^	0.780	0.865	0.854	0.847	0.710	0.806
	Best2^*c*^	0.725	0.863	0.855	0.845	0.799	0.791

Next, we analysed if the combination of different similarity measures would improve the results using just one single measure. As expected, indiscriminate averaging of all available measures (Consensus/All, Table 5) gave worse results than the best measure in each hierarchy level, since RMSD values were included, shifting the average to lower values. Selecting the best measure of each similarity comparison method (e.g. CE/Z, DaliLite/Z, FAST/SN, MaxCMO/Overlap, TM-align/TM, and USM/USM in the *Fold *level) led to an overall improved performance for all but the *Class *levels. A further reduction of the contributing methods, selecting the *three *best ones, showed an unexpected result. In all but the *Superfamily *and the *Protein *levels, the performance of the Consensus/Best3 method was better, having a greater AUC value than any of the single methods (compare Figure 6). The same synergistic effect was achieved for the two exceptions mentioned by forming the consensus from the *two *methods with the highest AUC values. The reason for this synergistic effect lies in an improved ranking of the pairs of proteins, having obtained similarity values that better discriminate between "similar" and "dissimilar".

These synergistic effects prove that ProCKSI's new Consensus measure can outperform even well established and reliable similarity comparison measures like DaliLite and CE. Hence, in order to maximise this synergy, finding the optimal combination of different similarity methods is crucial.

### Benchmark Tests

In the previous sections, we have shown the influence of different similarity methods and measures on the *quality *of the results. Here, we concentrate on their *speed *(including pre- and post-processing times) in order to show ProCKSI's performance. We have conducted benchmark tests and give the calculation times for the structure comparisons using five different datasets with different numbers of protein chains with six different similarity methods (Table 6 and Table 7). Additionally, the time for the preparation of the contact maps needed for the USM and MaxCMO calculations are given. These include times to parse the PDB files, extract the C_*α *_or C_*β *_atoms representing the protein structure, and calculate the distance matrix and contact map for each structure.

**Table 6:** Benchmark Tests of ProCKSI: Datasets. Overview of the datasets used for the benchmark tests of ProCKSI, comprising ^*a *^the first chain of the first model, and ^*b *^all chains of the first model, respectively. The average number of residues per chain is rounded to the next integer value. The hash symbol (#) abbreviates *Number of*.

Dataset		# Chains per Dataset	# Comparisons per Dataset	# Residues per Dataset	# Residues per Chain
CK34	[17]^*a*^	34	595	6102	179
CK53	[17]^*b*^	53	1431	9939	188
PK45	[83]^*a*^	45	1035	13360	297
PK49	[83]^*b*^	49	1225	12977	270
LKR6	[18]^*a*^	6	21	2296	383
LKR15	[18]^*b*^	15	120	4740	339
RS119	[87]^*a*^	119	7140	23053	197
RS212	[87]^*b*^	212	22578	39399	198
S33	[21]^*a*^	33	561	5532	168
S73	[21]^*b*^	73	2701	12999	178

**Table 7:** Benchmark Tests of ProCKSI: Calculation Times. Calculation times of all datasets with different similarity comparison methods used for the benchmark tests of ProCKSI. For USM and MaxCMO, the calculation times include the preparation times for the contact maps (CM) needed. The datasets are defined in Table 6

Dataset	Times [min] for						
	CM	USM	FAST	TM-align	DaliLite	CE	MaxCMO
CK34	0.48	1.72	3.20	2.08	19.95	16.78	81.70
CK53	0.73	4.23	5.07	4.97	50.93	48.42	210.23
PK45	1.08	5.83	11.93	7.90	107.67	69.03	471.70
PK49	1.10	7.20	9.62	7.68	112.65	66.62	470.52
LKR6	0.18	1.20	1.23	1.00	3.90	1.40	19.43
LKR15	0.42	1.93	1.58	1.40	14.60	6.70	70.27
RS119	1.77	17.18	17.15	28.75	210.63	305.95	1111.62
RS212	3.15	47.18	50.07	83.05	613.70	867.52	3275.48
S33	0.37	1.93	1.73	1.73	22.02	8.73	76.90
S73	0.83	4.88	6.57	8.17	124.43	51.87	419.03

**Figure 7:**
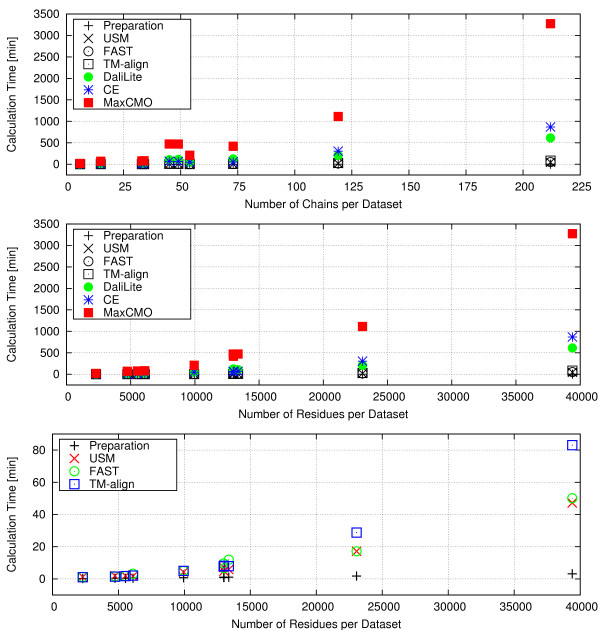
**Benchmark Tests of ProCKSI**. ProCKSI's response times for the completion of a request using all available similarity measures as a function of the number of all chains (top) and the number of residues (middle and bottom) in the corresponding dataset. The bottom panel is a magnification of the middle one, displaying only the fastest three methods and the contact map preparation for more clarity.

The benchmark tests were performed on our mini cluster with 3 dual-processor Intel Xeon/3.2 GHz computers with 4 GB memory. The independent similarity comparison modules were distributed in parallel over all available cluster nodes, making sure that the USM and MaxCMO calculations started not before the contact map preparations had finished. Figure 7 shows ProCKSI's response times for the completion of a request as a function of the size of the corresponding dataset, being represented as the dataset's total number of residues (Figure 7 – middle and bottom). Instead of using the number of chains per dataset only (Figure 7 – top), this measure reflects the size of the entire dataset better, also including the different protein sizes (Table 7).

The USM calculations are almost always the fastest to produce similarity values for an all-against-all structure comparison, but do not give an alignment. For smaller datasets, USM is closely followed by TM-align, whereas FAST beats TM-align for the biggest dataset (RS212). For the latter, performing over 22500 comparisons, USM and FAST return the complete results in about 50 minutes, and TM-align takes less then 85 minutes. This is still more than seven times faster than DaliLite, and ten times faster than CE. The slowest method in our benchmark test is the MaxCMO method, which took over two days to complete all comparisons. MaxCMO is implemented as a randomised heuristic algorithm hence requiring for each pair of structures to be compared several restarts as to gain statistical confidence. We used 10 restarts in this benchmark test resulting in up to five times higher calculation times than those of DaliLite. As mentioned before, a higher restart factor provides better chances of getting a better alignment but consumes more computation time.

These benchmark tests show clearly that the time scales the different similarity comparison methods operate on can be quite different. Thus, when producing a consensus similarity result it is important to take both *time *and *quality *into account as one might get a reasonable good result with the right combination of fast and reliable comparison methods.

## Conclusion and Discussion

### Summary

In this paper, we have introduced a new decision-support meta-server for ***Pro****tein (Structure) ****C****omparison*, ***K****nowledge*, ***S****imilarity and ****I****nformation *(ProCKSI). We have conducted three different experiments with different datasets in order to verify ProCKSI's new *Consensus *method based on a total-evidence approach. In a first test, we evaluated results from the CASP6 competition using the ProCKSI/Consensus method, which included all similarity comparison measures using contact maps as their input (USM, MaxCMO/Overlap, MaxCMO/Align). ProCKSI's new Consensus method agrees very well with CASP's GDT-TS method, the community's gold standard. In the few cases where the two methods produced contradictory ranking results, the ProCKSI/Consensus method could detect a better model with a higher similarity (value) and even higher sequence similarity.

In our second experiment, we tested the influence of different combinations of similarity measures on the clustering result of a set of protein kinases. The results using structural similarity comparison methods were compared against the classification scheme by Hanks and Hunter that had been derived from sequence similarities. Again, confirming the findings of our first experiment, none of the similarity methods completely reproduced the original classification when taken separately. ProCKSI's Consensus method, on the other hand, using just USM and DaliLite/Z, was able to reproduce the correct clustering according to Hanks and Hunter.

In our third experiment, we analysed the quality and performance of six different protein comparison methods as provided by ProCKSI by means of *Receiver Operator Characteristics *(ROC). Using the Rost and Sander dataset, the *Area Under the Curve *(AUC) values were employed in order to compare the results against SCOP's hierarchical classification levels as the gold standard. We investigated different combinations and sets of similarity measures in order to produce the best consensus measure. Surprisingly, combining the best *three *measures for SCOP's Class, Fold, Family and Species levels, or the best *two *measures for the remaining levels, respectively, we obtain higher AUC values than *any *of the contributing similarity measures by themselves. This synergistic effect shows that ProCKSI's new Consensus measure can outperform for some datasets even well established and reliable similarity comparison measures such as DaliLite and CE.

Additionally, we also benchmarked ProCKSI on various other datasets in terms of compute time, and found it competitive with current state of the art.

### Discussion

ProCKSI implements several different similarity methods and allows the user to provide results from his/her own similarity assessment, which are treated equally to ProCKSI's own results. When trying to produce a consensus similarity result it is important to take both *time *and *quality *into account as one might get a reasonable good result with the right combination of fast and reliable comparison methods. For example, other web servers for protein structure comparison (e.g. DALI, CATH, LGA, CE, FATCAT, FAST, etc.) allow only *pairs *of proteins to be compared or they compare *one *given protein structure against a database of pre-calculated/pre-aligned structures. In the latter case, the result of such a comparison might be delivered almost instantaneously, whereas the response time for a pairwise comparison or arbitrary proteins depends on the following factors: a) the algorithms used to make the comparison, b) the sizes of the proteins to be compared, and c) the servers' load and internet traffic. When comparing a set of *N *proteins against each other, there are N2+N2
 MathType@MTEF@5@5@+=feaafiart1ev1aaatCvAUfKttLearuWrP9MDH5MBPbIqV92AaeXatLxBI9gBaebbnrfifHhDYfgasaacH8akY=wiFfYdH8Gipec8Eeeu0xXdbba9frFj0=OqFfea0dXdd9vqai=hGuQ8kuc9pgc9s8qqaq=dirpe0xb9q8qiLsFr0=vr0=vr0dc8meaabaqaciaacaGaaeqabaqabeGadaaakeaadaWcaaqaaiabd6eaonaaCaaaleqabaGaeGOmaidaaOGaey4kaSIaemOta4eabaGaeGOmaidaaaaa@3203@ different combinations of pairs to be calculated, assuming that the comparison of protein *p*_1 _with protein *p*_2 _gives the same result as comparing *p*_2 _with *p*_1_.

In addition to the algorithms' complexity and the number of protein pairs to be compared when calculating the similarity of a set of proteins with a specific comparison server that allows only pairwise comparisons, each pair has to be generated and uploaded separately, and the desired models and chains have to be selected/extracted repeating this procedure for the same protein file more than once. After submitting the job, it has to be checked periodically until results are available, which then must be downloaded separately. Finally, the results would have to be integrated manually in order to produce a similarity matrix for all proteins in the set. This can be tedious and error prone, especially when dealing with sets of tens or hundreds of structures. ProCKSI, on the other hand, helps to minimise the data management overhead by preparing the entire dataset once in a few steps, by giving access to a variety of similarity methods and measures in one easy-to-use interface, by keeping track of the progress of all calculations, and by seamlessly and automatically integrating all results. That is, ProCKSI hides from the end user the complexity behind a systematic comparison studies.

As our experiments have shown, not all comparison methods perform equally well on all datasets. MaxCMO, for instance, gave excellent results in our CASP experiment, but could discriminate the Kinases only partially. The important lesson here is not that MaxCMO performed poorly on the Kinases dataset (as we mentioned in the introduction that every method has an Achilles heel), but rather that even when adding to the consensus a method that discriminates the dataset fairly poorly, one can still obtain comparably good results. These findings lend support to our integrative approach of combining various similarity measures thus producing a *robust *consensus similarity, and show that the best results potentially do prevail even when adding "noise" to the data. This is a particular relevant observation as in general the biologist, faced with a given dataset, does not know *a priori *which method to use. Hence, he/she would be on safer grounds if he/she was to use *all *of the available methods (through a decision support system such as ProCKSI) and rely on a consensus method.

We have also found that there are different optimal combinations of different methods when generating the consensus similarity picture for different datasets. Hence, finding a good set and combination of similarity comparison methods for a given dataset remains a key open question.

## Future Work

In the future, we plan to extend ProCKSI integrating other similarity methods and link to further databases, e.g. [94,95], and systematically investigate the impact of different compressors in the USM [96].

In order to cope with the vast amount of calculations and data, we will seek to enhance our computational platform by recruiting more compute servers, by utilising established web services for protein comparison, and by deploying the calculations to the GRID.

More importantly, we will investigate new and more intelligent ways of computing consensus similarities using e.g. machine learning techniques [97], and integrate automated cluster validation techniques, e.g. [98,99]. A measure of variance such as averaged ROC curves from bootstrapping or cross-validation with a variety of different datasets is needed in order to give a final conclusion about the optimal set of comparison methods [86]. This at hand, we will be able to give the user more and better advice and guidelines of which methods to use for a particular problem.

Additionally, we plan to integrate into ProCKSI a second analysis strategy using average consensus trees and supertrees [100,101] so as to complement our current total-evidence approach [47,102,103].

## Availability and Requirements

**Project name: **ProCKSI

**Project home page: **

**Operating system(s): **Linux (back-end), platform independent (front-end)

**Programming languages: **PERL, Java, C++

**Other requirements: **Web Browser, Java Runtime Environment (JRE), JavaScript, Cascading Style Sheets (CSS)

**License: **Web server freely available without registration

**Restrictions to use by non-academics: **on request

## List of Abbreviations

AUC : Area Under the Curve;

CASP : Critical Assessment of Techniques for Protein Structure Prediction;

CE : Combinatorial Extension of the optimal path;

CL : Complete Linkage;

CM : Contact Map;

DALI : Distance Matrix Alignment;

DM : Distance Matrix;

FAST : FAST Alignment and Search Tool;

FPr : False Positive rate;

GDT-TS : Global Distance Test – Total Score;

HH : Hanks and Hunter;

LGA : Local Global Alignment;

MaxCMO : Maximum Contact Map Overlap;

NCD : Normalised Compression Distance;

PDB : Protein Data Bank;

PK : Protein Kinase;

PKR : Protein Kinase Resource;

ProCKSI : Protein (Structure) Comparison, Knowledge, Similarity and Information;

RMSD : Root Mean Square Distance;

ROC : Receiver Operator Characteristics;

SCOP : Structural Classification Of Proteins;

SDA : Sequence Dependent Analysis;

SIA : Sequence Independent Analysis;

SL : Single Linkage;

SM : Similarity Matrix;

SSM : Standardised Similarity Matrix;

TM : Template Modelling;

TPr : True Positive rate;

UPGMA : Unweighted Pair Group Method with Arithmetic mean;

USM : Universal Similarity Metric;

VRML : Virtual Reality Markup Language;

WMV : Ward's Minimum Variance.

## Authors' contributions

DB: software design, implementation, writing, assessment. NK: project conception, software design, writing, assessment, funding. JDH: writing, discussions, funding. JB: writing, discussions. EKB: writing, funding. All authors read and approved the final manuscript.
